# Emerging Concepts of Data Integration in Pathogen Phylodynamics

**DOI:** 10.1093/sysbio/syw054

**Published:** 2016-06-06

**Authors:** Guy Baele, Marc A. Suchard, Andrew Rambaut, Philippe Lemey

**Affiliations:** 1 *Department of Microbiology and Immunology, Rega Institute, KU Leuven - University of Leuven, Leuven, Belgium*; 2 *Department of Biomathematics, David Geffen School of Medicine, University of California, Los Angeles, CA 90095, USA*; 3 *Department of Human Genetics, David Geffen School of Medicine, University of California, Los Angeles, CA 90095, USA*; 4 *Department of Biostatistics, School of Public Health, University of California, Los Angeles, CA 90095, USA*; 5 *Institute of Evolutionary Biology, University of Edinburgh, Kings Buildings, Edinburgh EH9 3FL, UK*; 6 *Centre for Immunity, Infection and Evolution, University of Edinburgh, Kings Buildings, Edinburgh EH9 3FL, UK*

## Abstract

Phylodynamics has become an increasingly popular statistical framework to extract evolutionary and epidemiological information from pathogen genomes. By harnessing such information, epidemiologists aim to shed light on the spatio-temporal patterns of spread and to test hypotheses about the underlying interaction of evolutionary and ecological dynamics in pathogen populations. Although the field has witnessed a rich development of statistical inference tools with increasing levels of sophistication, these tools initially focused on sequences as their sole primary data source. Integrating various sources of information, however, promises to deliver more precise insights in infectious diseases and to increase opportunities for statistical hypothesis testing. Here, we review how the emerging concept of data integration is stimulating new advances in Bayesian evolutionary inference methodology which formalize a marriage of statistical thinking and evolutionary biology. These approaches include connecting sequence to trait evolution, such as for host, phenotypic and geographic sampling information, but also the incorporation of covariates of evolutionary and epidemic processes in the reconstruction procedures. We highlight how a full Bayesian approach to covariate modeling and testing can generate further insights into sequence evolution, trait evolution, and population dynamics in pathogen populations. Specific examples demonstrate how such approaches can be used to test the impact of host on rabies and HIV evolutionary rates, to identify the drivers of influenza dispersal as well as the determinants of rabies cross-species transmissions, and to quantify the evolutionary dynamics of influenza antigenicity. Finally, we briefly discuss how data integration is now also permeating through the inference of transmission dynamics, leading to novel insights into tree-generative processes and detailed reconstructions of transmission trees. [Bayesian inference; birth–death models; coalescent models; continuous trait evolution; covariates; data integration; discrete trait evolution; pathogen phylodynamics.

Grenfell et al. (2004) originally introduced phylodynamics to describe “the melding of immunodynamics, epidemiology and evolutionary biology.” Grown into a mature field that aims to enhance our understanding of infectious disease transmission and evolution, phylodynamics relies on phylogenetic inference as the core analytical tool to recover evolutionary and epidemic processes from the mutations that accumulate in the genomes of rapidly evolving pathogens during spread of an epidemic. These mutations may confer phenotypic differences that allow viruses to infect different cell types, to evade host immune responses or to transmit by different routes, hosts or vectors (Holmes et al. 1995), but the mutations may also represent the molecular footprint of epidemiological processes that can otherwise not directly be observed. Extracting such information from genetic data represents the primary goal of phylodynamics and requires the integration of additional data and models in a phylogenetic framework. Therefore, phylodynamics is not only considering the interplay between evolution and epidemiology from a conceptual stance, but the integration is made concrete through advances in statistical modeling and computational inference, a key focus of this review.

Rapidly evolving pathogens are unique in that their ecological and evolutionary dynamics occur on the same timescale and can therefore potentially interact. Time of sampling therefore represents important information to incorporate in phylodynamic analyses because it allows calibration of phylogenies, and hence epidemic histories, of rapidly evolving pathogenies in calendar time units (Pybus and Rambaut 2009). Molecular clock models that formalize the relationship between sequence divergence and evolutionary time have been extended specifically for this purpose, and models accommodating sampling time now represent the cornerstone of time-measured phylodynamics (Rambaut 2000; Shapiro et al. 2011). Populations from which “heterochronous” sequence data can be obtained are colloquially referred to as measurably evolving populations (MEPs) (Drummond et al. 2003), a concept that does not only apply to rapidly evolving pathogens but also extends to populations from which ancient DNA can be sampled (e.g., Hofreiter et al. (2001); Päabo et al. (2004); Shapiro et al. (2004); Molak et al. (2015)). Not surprisingly, location of sampling has also received a great deal of attention because infectious disease transmission is an inherently spatial process. However, location in an epidemic network is not necessarily determined by geographical position, but may be more appropriately represented by position in a social or sexual network, the proximity to vector breeding sites, the movement of hosts or infectious agents through commerce, air travel, wind, or other factors (Smith 2005; Brockmann and Helbing 2013). By offering explicit patterns of connectivity in infectious disease populations, genetic data can be instrumental in determining the importance of these factors in pathogen spread.

Together with spatial processes, the intensity of transmission and growth or decline in epidemic size can also leave an imprint in viral genomes. This has led to the widespread application of models that relate patterns of evolutionary ancestry to parameters quantifying population size changes or genealogical branching rates. Population genetic approaches based on the coalescent have enjoyed sustained popularity as “tree-generative” models, with the coalescent describing the relationship between the demographic history of a large population and the shared ancestry of individuals randomly sampled from it, as represented by a genealogical tree. This relationship is formalized by a probability distribution of times between the coalescence events in the sample genealogy, which depends on a demographic function that describes population-size change through time. Initially focusing on specific parametric functions of effective population size change through time (Pybus et al. 2000; Strimmer and Pybus 2001), coalescent modeling has evolved to allow for populations with stochastically varying sizes through the use of flexible nonparametric modeling of demographic history (Drummond et al. 2006b; Minin et al. 2008; Gill et al. 2013).

Birth–death models trace back to the work of Kendall (1948) and describe a stochastic process that typically starts with a single species, and allows species to give birth to a new species after an exponential waiting time or to die after an exponential waiting time. Calculation of the probability density of a genealogy generated by the birth–death process has been developed for complete sampling (Gernhard 2008) and incomplete sampling (Stadler 2010) of the population. The latter requires a combination of the birth–death process with a model of the sampling process (Volz and Frost 2014), which can be assumed to vary through time (Stadler et al. 2013).

**Figure 1.**
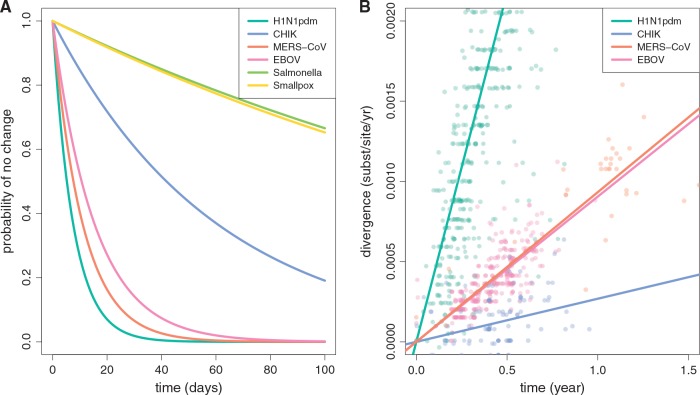
Temporal signal in pathogen genomes sampled through time. A) Plot of the probability of observing no changes between two genomes of length ($N$) separated by the given number of days ($x$) for a given rate of evolution per site per year ($\mu$). We used $N = 13,100$ and $\mu = 0.0037$ for Influenza A/H1N1 (Stadler et al. 2013), $N = 30,100$ and $\mu = 0.0011$ for MERS-CoV (Cotten et al. 2014), $N = 19,800$ and $\mu = 0.0012$ for EBOV (Park et al. 2015), $N = 13,100$ and $\mu = 0.0005$ for CHIK, $N = 4,641,576$ and $\mu = 0.00000032$ for Salmonella Typhimurium DT104 (Mather et al. 2013), and $N = 190,000$ and $\mu = 0.0000082$ for Smallpox. An online application to plot such probabilities is available at http://epidemic.bio.ed.ac.uk/node/79. B. Root-to-tip divergences as a function of sampling time based on publicly available Influenza A/H1N1, MERS-CoV and EBOV genomes as well as for an unpublished CHIK data set. The regression plots were rescaled for each virus such that the oldest genome in each dataset was set at time = 0 and the regression line has zero divergence at time = 0.

Pathogen sequences sampled over time and space and their associated traits are now typically analyzed using Bayesian statistical approaches. Bayesian modeling and inference is particularly attractive for phylodynamics because it represents a natural framework for data integration and it also avoids the need to condition on data summary statistics and associated error propagation. By adequately taking into account the uncertainty of unobservables — for example the genealogy — when attempting to draw inference from processes giving rise to or unfolding on genealogies, Bayesian inference constitutes a general and coherent statistical framework that solely conditions on the observed sequences and associated information. Bayesian genealogical approaches can also naturally accommodate tree-generative process as tree priors. However, a Bayesian full probabilistic model requires the specification of appropriate prior distributions over all the parameters in the model, which can make practitioners feel uncomfortable at times and calls for an examination of the sensitivity of posterior estimates with respect to choices in prior distribution. MrBayes (Ronquist et al. 2012) and BEAST (Drummond et al. 2012) represent two popular Bayesian inference packages in the field that offer a wide range of evolutionary and/or population genetic models, but we restrict ourselves to the BEAST framework in this review because of its traditional focus on measurably evolving pathogens and time-measured trees.

The increasing availability and quality of viral genome sequences, the growth in computer processing power, and the development of sophisticated statistical methods have all contributed to the current popularity of Bayesian phylodynamic inference in infectious disease research (Pybus and Rambaut 2009). However, adequately informing particular evolutionary and population genetic models from genetic data in isolation can be challenging. This may be particularly problematic for trait evolutionary models that are generally fitted to only a single observation of the trait associated with molecular sequences. Parameter uncertainty can hamper the establishment of definite associations with external covariates, and more generally, efficient hypothesis testing. A promising avenue of research that currently emerges is the integration of covariates or other data, like time series of case reports, in the evolutionary and population genetic reconstructions. This represents an additional level of data integration that offers many advantages.

In this review, we first highlight modern approaches to integrate trait and sequence evolution in pathogen phylodynamics and discuss an example of both discrete and continuous trait reconstruction. We expand on this by highlighting several applications that are not restricted to spatial problems. We subsequently discuss how potential covariates of sequence and trait evolutionary processes can be integrated and how additional information about epidemic dynamics can be incorporated in analyses that serve both to reconstruct and test hypotheses about phylodynamic history. We conclude this review by discussing recent developments and future perspectives in the field of phylodynamics.

## Sequences and Traits

In terms of the sequence evolutionary process, we make a distinction between the mode of evolution or the relative intensities by which sequence characters are exchanged in evolutionary history and the tempo of evolution which determines how this exchange process scales in units of time. The former can be modeled by different parameterizations of continuous-time Markov chain (CTMC) models and for which we refer to the general phylogenetic literature (Felsenstein 2004; Yang 2006; Lemey et al. 2009b). For the latter, time of sampling provides the most important source for calibration in phylodynamic inference.

As mentioned above, molecular clock models allow quantification of the rate of substitution, and for MEPs, their calibration is generally based on the divergence that accumulates over the sampling time range. The extent to which we expect to observe genetic changes between viral sequences sampled at different times will depend on the overall rate of substitution (and its constancy), the length of the sequences that can be obtained and the differences in time of sampling. We illustrate this for complete genome sampling from four different viral pathogens that have caused relatively recent outbreaks: pandemic influenza A (A(H1N1)pdm09), Middle East respiratory syndrome coronavirus (MERS), Chikungunya virus and Ebola virus (Fig. 1). For comparison, we also include a DNA virus (smallpox) and a bacterial pathogen (Salmonella Typhimurium DT104). These pathogens differ both in genome length and short-term rates of substitutions, which leads to different expectations for observing changes as time elapses between sequence samples (Fig. 1A). Influenza A(H1N1)pdm09 genomes are expected to accumulate more changes over time, closely followed by MERS and Ebola genomes, whereas this occurs at a considerably slower rate for Chikungunya but still faster than smallpox and Salmonella. The divergence accumulating in actual sequence data sampled during outbreaks of these viruses is largely consistent with the respective probability plots (Fig. 1B). Further in line with these expectations and empirical patterns, Hedge et al. (2013) have been able to accurately estimate evolutionary and epidemiological parameters from A(H1N1)pdm09 genomes as early as two months after the first reported case, which demonstrates a clear potential for real-time phylodynamic characterization during emerging epidemics. In general, assessing whether viral sequence samples contain sufficient temporal signal is a cautionary — if not a necessary — step prior to fitting dated-tip molecular clocks in order to extract epidemiological processes from viral sequences.

As in the general field of molecular evolution, making a molecular clock assumption serves two main purposes in phylodynamics: dating the phylogenetic history and providing a mechanistic description of the evolutionary process. The former is particularly useful to date cross-species transmissions, viral outbreaks, and the historical spread of epidemics. At a smaller scale, evolutionary estimates may also help to pinpoint the individual’s infection time or assess transmission hypotheses (Vrancken et al. 2014b). Furthermore, incorporating sampling time can assist in adequately rooting pathogen phylogenies, which in turn may help in resolving the evolutionary origins of emerging viruses (e.g., for Ebola: Baize et al. (2014); Dudas and Rambaut (2014)). Coupled to tree-generative processes, time-measured genealogies also enable estimation of epidemic growth rates per unit time or basic reproduction rates, even early in a pandemic (Fraser et al. 2009; Volz et al. 2013; Hedge et al. 2013).

The nature by which the temporal information contained in the sampling times can be connected to the genetic similarities embedded in the sequences depends on the specific formulation of the molecular clock hypothesis. The original constant or “strict” molecular clock model, postulating a single rate of substitution across all branches in the phylogeny, has proven to be too restrictive in many applications and can mislead divergence date estimation as well as phylogenetic inference (Yoder and Yang 2000; Ho and Jermiin 2007). This has motivated several developments to relax the strict molecular clock assumption, which can be largely subdivided into models that allow a limited number of discrete rate changes in the phylogeny (“local” molecular clocks, Yoder and Yang (2000)) and models that allow the rate to change in a continuous fashion by either assuming or not assuming a relationship between the rates on ancestral and descendent branches (autocorrelated (Thorne et al. 1998) and uncorrelated (Drummond et al. 2006a) relaxed clocks respectively). Whereas the uncorrelated relaxed clock approach has gained considerable attraction as a generic clock modeling approach, it is worth noting that local molecular clock modeling also has interesting applications in pathogen phylodynamics. For example, assuming local molecular clocks for host-specific lineages can lead to more accurate reconstructions of phylogenetic history and this resulted in far more consistent evolutionary reconstructions across different segments of influenza A (Worobey et al. 2014). Host-specific rates of evolution also represent a particular scenario of local molecular clock modeling, but when such a specific hypothesis is not available a priori, a restricted number of discrete rate changes can still be identified through a random local molecular clock approach (Drummond and Suchard 2010).

When the aim is to estimate divergence times, molecular clock specification may be considered as a nuisance and Bayesian model averaging approaches are particularly attractive in this context (Li and Drummond 2012). Accurately modeling substitution variation becomes a primary interest when the tempo of evolution is scrutinized as a mechanistic process. Understanding the sources of rate variation, and in particular determining the role of host ecology, will be the major focus of our discussion on covariates for evolutionary processes. In the next section, we first outline how additional data can be integrated with genetic data through trait evolutionary modeling, both for discrete and continuously valued traits.

## Discrete Trait Evolution

Examining traits associated with sequence data in an evolutionary context requires a model of how the traits evolve throughout phylogenetic history. Discrete trait modeling can take guidance from standard phylogenetics and borrow the process of exchange between sequence character states as a generic model for how traits substitute their state over tree branches. Arguably the most frequently considered traits in phylodynamics, and molecular evolution in general, are spatial locations. The interest in spatial dispersal, migration, or vicariance goes back to early naturalists studying the geographic distribution of species, even in the absence of genetic data (Haeckel 1866), and has developed into its own research field referred to as phylogeography. This field has witnessed several developments for reconstructing the geographic locations of ancestral lineages (e.g., Bloomquist et al. 2010), but here we focus on stochastic models of phylogenetic diffusion models and the structured coalescent.

The reconstruction of discrete character states on a phylogeny has traditionally relied on the principle of parsimony, which aims at minimizing the number of historical character changes required to produce the states of the trait we observe at the tree tips (e.g., (Maddison et al. 1984)). As in standard phylogenetics, maximum likelihood (ML) has been proposed as a probabilistic alternative to ancestral reconstruction. Using phylogenetic CTMC modeling, Pagel (1999b) introduced an ML approach to infer ancestral character states for binary discrete characters, which can be readily generalized to multistate characters (Pagel et al. 2004). As phylogenies are seldom known with certainty and generally represent the result of reconstruction procedures, Pagel et al. (2004) describe a general procedure for reconstructing ancestral character states across a statistically justified sample of trees estimated by a prior Bayesian phylogenetic analysis. This procedure combines information about the uncertainty of the phylogeny with uncertainty in the estimate of the ancestral state and avoids constraining the sample of trees to only those that contain the ancestral node or nodes of interest, which can lead to overconfidence in a particular reconstruction. A more efficient, simulation-free method for inferring ancestral traits is presented by Minin and Suchard (2008).

In the context of biogeography, Sanmartín et al. (2008) have also adopted Bayesian inference of discrete phylogenetic diffusion processes, this time proposing to jointly estimate the phylogeny and the trait evolutionary process (in MrBayes, Ronquist et al. (2012)). As discrete diffusion models, the authors consider the most general CTMC process with a different rate of exchange for each pair of states — akin to a General Time-Reversible (GTR) model in nucleotide space (Tavaré 1986) — and constrained versions thereof that represent specific scenarios of the island biogeography process they aim to characterize. A similar Bayesian full probabilistic connection between sequences and traits has also been implemented in BEAST, which initially focused on spatiotemporal reconstructions of viral spread (Lemey et al. 2009a). These sister approaches offer extensive modeling flexibility, but at the expense of a quadratic growth in number of instantaneous rate parameters in the CTMC matrix as a function of the state dimensionality of the trait. This is particularly problematic because the rate parameters are only informed by a single trait character observed at the tree tips.

The specific island biogeography application by Sanmartín et al. (2008) focused on a manageable problem by considering only a restricted number of spatial groups and by sharing a single rate matrix across different groups of organisms with independent phylogenies. However, Bayesian inference also offers specific ways to protect against over-parameterization. Prior specification is an obvious one that was exploited in viral epidemiology (Lemey et al. 2009a), for example, by simply proposing higher rates of diffusion between nearby locations a priori. Motivated by the argument that only a limited set of state transitions throughout evolutionary history can leave their trace in the distribution of a single character, Lemey et al. (2009a) adopted Bayesian stochastic search for variable selection (BSSVS) to reduce the number of rate parameters to a restricted set that provides the most adequate parsimonious description of the diffusion process. In the context of discrete diffusion models, BSSVS associates every pairwise rate with a binary indicator but a priori prefers to only invoke a minimal number of rates with an nonzero indicator to explain the tip trait observations. Variable selection and informative prior specification both increase statistical efficiency, which becomes even more important when drawing inference from sparse data under more complex models, for example, assuming two different rates of diffusion for each directionality between a pair of states (Edwards et al. 2011).

Simultaneous inference of sequences and traits implies that both data sources can impact the phylogeny. In most cases, however, this is of little practical importance because the information contained in the multiple sites comprising the sequence alignment can swamp the information present in a single trait character. However, in the presence of very shallow sequence diversity, the tendency of taxa to cluster together by trait state may become more prominent. It is probably not unreasonable to assume that taxa with the same trait character will be more closely related, but the relative strength of such a statement may be subject of debate. It is worth noting that the simultaneous inference capability has been explicitly leveraged to obtain “combined-data” trees in specific contexts, for example for morphological evolution (Nylander et al. 2004) and protein structure evolution (Scheeff and Bourne 2005).

**Table 1.** Discrete trait applications

Trait	Organism	References
Spatial	Avian Influenza A H5N1	Lemey et al. (2009a)
	Mycobacterium tuberculosis	Comas et al. (2013)
	Influenza A H3N2	Lemey et al. (2014)
Spatial and temporal (season)	Influenza A H3N2	Bahl et al. (2011)
Host	Bat rabies	Streicker et al. (2010, 2012); Faria et al. (2013)
	Staphylococcus aureus CC398	Ward et al. (2014)
	Campylobacter	Dearlove et al. (2015)
	Salmonella Typhimurium DT104	Mather et al. (2013)
Antibiotic resistance genes	Staphylococcus aureus CC398	Ward et al. (2014)
Virulence determinants	Staphylococcus aureus CC398	Ward et al. (2014)
HA & NA subtypes (reassortment)	Avian Influenza Virus	Lu et al. (2014)
Antigenic clusters	Influenza A H3N2	Zinder et al. (2013)
Animal tissue	Small ruminant lentivirus (SRLV)	Ramírez et al. (2012)
Larval characters	Thecostraca	Pérez-Losada et al. (2012)
Gag and env subtypes (recombination)	HIV-1 group M	Ward et al. (2013)
Leaf traits	Triodiinae	Toon et al. (2015)
Morphological characters	Pradosia	Terra-Araujo et al. (2015)
	Pycnandra	Swenson et al. (2015)
Habitat preference	Pradosia	Terra-Araujo et al. (2015)
Phyllotaxy	Polygonatum	Meng et al. (2014)
Habitat types	Mus Nannomys	Bryja et al. (2014)
Cellular compartments	HIV-1	Cybis et al. (2013)

*Notes:* A non-exhaustive overview of applications of the discrete trait modeling approach by Lemey et al. (2009a), demonstrating the general applicability of the methodology beyond the inference of viral phylogeographic processes.

**Figure 2.**
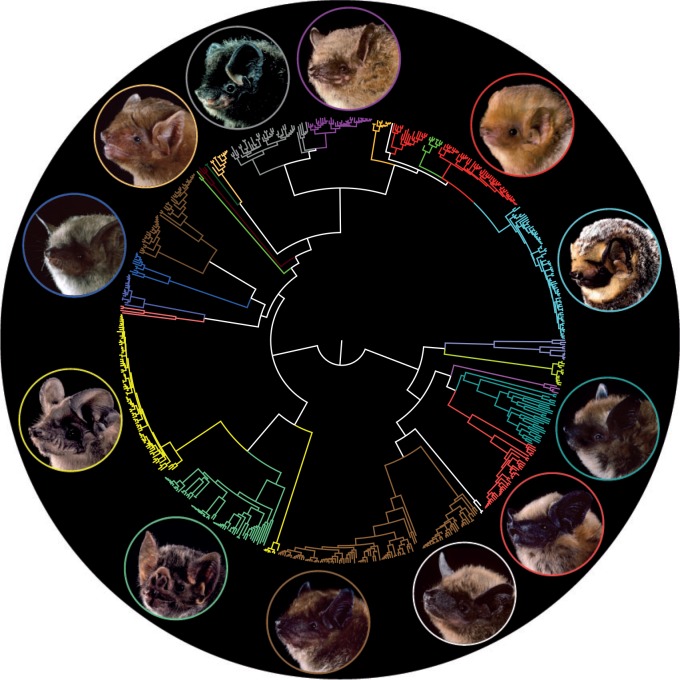
The multihost transmission dynamics of rabies in American bat species. Streicker et al. (2010) identified 18 phylogenetic lineages of rabies virus that were statistically compartmentalized to particular bat taxa. These lineages are represented by differently colored clades in the phylogeny, along with a selection of bat species involved. CSTs are defined as jumps to bat species different from the dominant species within each lineage or clade. These dynamics have been quantified though structured coalescent approaches (Streicker et al. 2010). Host switches on the other hand are defined as the jumps along internal branches to new hosts followed by successful transmission in the new host species. Inferring the history of host jumping along the branches (mostly represented by white branches) has been the subject of discrete trait reconstruction (Streicker et al. 2010). We thank Daniel Streicker for providing the tree and MerlinTuttle.org for granting permission to use the bat portraits.

Despite the overwhelming interest in geographic spread, various traits are now the subject of ancestral reconstruction analyses. To illustrate this, we present a sample of applications of the BEAST discrete trait modeling approach in Table 1, mostly focusing on nonspatial studies. These applications range from the reconstruction of morphological characters in animals and plants to antigenicity in influenza viruses and host species in pathogens and bacteria. In phylodynamics, host traits may be of particular interest as demonstrated by a seminal study on bat rabies viruses in the Americas (Streicker et al. 2010). Bat rabies in the Americas represents an interesting multihost system, in which rabies viruses jump between different bat hosts, occasionally resulting in sustained transmission in the new host species. Over time, this process has produced a phylogenetic structure comprising lineages that are compartmentalized by a dominant bat host (Fig. 2). To study the ancestral host shifts giving rise to this pattern, Streicker et al. (2010) applied Bayesian phylogenetic inference to demonstrate that these events have the tendency to occur between more closely related bat species. We will return to this example when discussing the integration of covariates in sequence and trait evolutionary processes to test the causes and consequences of this host switching process. The study by Streicker et al. (2010) also highlights an alternative approach to discrete trait diffusion because the cross-species transmission (CST) dynamics within each host-associated lineage were quantified using a structured coalescent approach. By factoring in the diversity within each host, this approach allowed them to estimate *per capita* CST rates and test their association with several potential explanatory variables.

The structured coalescent also emerged in the context of spatial migration and goes back to the seminal work by Hudson (1990) and Notohara (1990). As an extension of Kingman’s coalescent (Kingman 1982), the structured coalescent model builds on the standard Wright-Fisher model by specifying a number of discrete subpopulations, allowing location to explicitly affect the coalescent rate (Vaughan et al. 2014). Spatial subdivision into a number of distinct “demes” represents an obvious population structure, but as illustrated by the bat rabies example, any logical categorization of individuals can be considered by this model, including for example the temporal subdivision used in compartmental epidemiological models (discussed later in this review).

Probabilistic ancestral reconstruction and the structured coalescent rely on a very different set of assumptions, and have traditionally been interested in different estimates (ancestral states vs. population migration rates). Computational restrictions may have prevented the widespread adoption of structured coalescent approaches in phylodynamics. Interestingly, this is now being addressed by recent developments in Bayesian inference under the structured coalescent. Vaughan et al. (2014) introduced a new set of MCMC transition kernels in BEAST that achieve significantly faster mixing compared with previous transition kernels (Ewing et al. 2004). Further, De Maio et al. (2015) tackle the impractical computational demands when confronted with large numbers of subpopulations and migration events by introducing a new model-based approach that achieves a close approximation to the structured coalescent, while still integrating over all possible migration histories. Their Bayesian structured coalescent approximation (BASTA) combines the accuracy of methods based on the structured coalescent with computational efficiency, offering less biased estimated compared to discrete trait reconstruction approaches. De Maio et al. (2015) attribute this to specific assumptions associated with modeling migration of lineages as a mutational process.

## Continuous Trait Evolution

Modeling evolution of continuous characters stems from the field of comparative biology that was challenged with the problem of comparing continuous traits, for example to assess their correlation, across a number of taxa. Standard correlations assume independent data, which is obviously violated for taxa traits due to their shared ancestry. Felsenstein (1985) proposed the “independent contrasts” approach to deal with such nonindependence. This approach assumes that the changes in two characters follow a Brownian motion process, implying that they are drawn from a bivariate normal distribution with some degree of correlation between the characters along each branch of a phylogeny. In addition, the variance of the distribution of change along each branch is assumed to be proportional to the time elapsed on that branch. This procedure uses a phylogeny to identify a set of mutually independent comparisons between pairs of species, pairs of nodes, or a node and a species (Pagel 1997). Grafen (1989) presents a generalization of the independent contrasts approach that allows for trait adjustment by species-specific covariates. This approach connects general linear modeling to phylogenetic data and has become known as phylogenetic regression in the field of comparative biology.

In the conventional continuous random-walk model, traits evolve in each instant of time with a mean change of zero and unknown and constant variance. Originally introduced as an approximation of sequence evolution (Edwards and Cavalli-Sforza 1964), phylogenetic Brownian random walks have mostly been applied to phenotypic traits in comparative biology (see e.g., Martins (1994)). Analogous to discrete trait modeling, both maximum likelihood and Bayesian approaches have been proposed to draw inference under such models (Schluter et al. 1997; Pagel 1999a; Lemmon and Lemmon 2008). In phylodynamics, a bivariate Brownian random walk was again initially applied in a phylogeographic setting (Lemey et al. 2010). The Bayesian implementation proposed by this work not only avoids conditioning on the usual data summaries, for example a fixed tree and branch lengths, but also relaxes the constant diffusion rate assumption implied by a constant-variance random walk. Specifically, variation in diffusion rates among branches was modeled by borrowing from uncorrelated relaxed clock models (Drummond et al. 2006a), that is by rescaling the variance of the random walk (or precision in a Bayesian terminology) along each branch using a scalar drawn independently and identically from an underlying distribution. This relaxed random walk (RRW) appeared to be critical to accommodate a very high degree of heterogeneity that can underlie the spatial spread of pathogens, as was the case for the West Nile virus invasion in the United States (Pybus et al. 2012). The same analysis also challenged standard inference under these models, which initially relied on sampling internal node realizations of the bivariate locations but proved to be inefficient in this case. Analogous to efficient likelihood computation for discrete traits (Felsenstein 1981), Pybus et al. (2012) pursued an approach that integrates out internal node states in the Bayesian framework (see also Freckleton (2012)), which makes applications to large datasets more accessible.

Although diffusion rate variation can be taken into account, a phylogeographic RRW process still relies on dispersal as a function of geographic distance (or distance in Euclidean space). This may be unrealistic for pathogens that exploit human mobility or trade (e.g., human seasonal influenza (Lemey et al. 2014) and swine influenza respectively (Nelson et al. 2015)) and modeling efforts have demonstrated that pathogen spread follows “effective distances” measured along complex transportation networks (Brockmann and Helbing 2013). Due to these limitations, future applications of continuous trait modeling phylodynamics may also focus more on other traits, such as pathogen phenotypes and infection traits. A limited sample of applications of the continuous diffusion framework in BEAST (Lemey et al. 2010) (Table 2), mostly focusing on nonspatial traits, indeed highlights the general use of this approach even beyond the field of biology.

**Table 2.** Continuous trait applications

Trait	Organism or data	References
Spatial	Chorus frogs	Lemmon and Lemmon (2008)
	Raccoon rabies	Lemey et al. (2010)
	Indo-European languages	Bouckaert et al. (2012)
	Asian Languages	Dunn et al. (2013)
	Ainu language	Lee and Hasegawa (2013)
Normal mode	Enzymes	Lai et al. (2012)
Antigenic measurements	human Influenza A and B	Bedford et al. (2014)
(Set point) viral load	HIV-1	Vrancken et al. (2014a)
	Sigma viruses	Vrancken et al. (2014a)
Antibody neutralization	HIV-1	Vrancken et al. (2014a)
Femur length	Theropod dinosaurs	Lee et al. (2014)
Shell lengths	Chemosymbiotic deep-sea mussels	Lorion et al. (2013)
Generation times	Anthropoids	Schrago (2014a,b)
Gene flow	Rhesus macaques	Strickland et al. (2014)

*Notes:* A nonexhaustive overview of the applications of the continuous trait modeling approach by Lemey et al. (2010), demonstrating the broad range of applications even going beyond biology. For example, their approach can be used to simultaneously use sequence data with antigenic measurements to estimate antigenic cartography for influenza or to infer the spatiotemporal expansion of language families.

For pathogens, traits related to phenotype or infection severity, for example, will generally be measured through experimental assays. Although this does not hamper the general application of multivariate diffusion models, it can impose additional modeling challenges. One of these challenges is adequately incorporating measurement error (analogous to intraspecific variation for organismal traits), that is conditioning on the data (the repeated measures) rather than data summaries such as their mean and variance. The Bayesian multivariate diffusion approach naturally achieves this by numerically integrating the unobserved average tip trait values based on repeated measures (Vrancken et al. 2014a).

**Figure 3.**
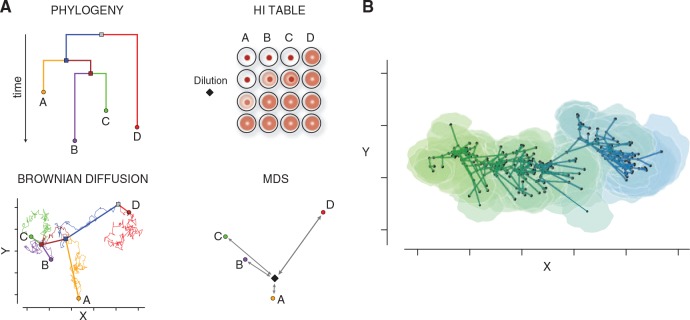
Antigenic cartography meets Brownian phylogenetic diffusion. A) Conceptual representation of the integration of genetic and antigenic evolution through a Bayesian MDS approach. In the HI assay (on the right), the antigenic phenotype is investigated by measuring the cross-reactivity of a virus (A, B, C, or D) strain to serum ($\blacklozenge$) raised against another strain. Based on these HI measurements, MDS approaches allow to position viruses in lower-dimensional space such that the distances in this space best fit the HI assay titres. A probabilistic interpretation of MDS assumes that the observed differences are centered around their cartographic expectation, in which case the virus locations are estimable parameters. We refer to Bedford et al. (2014) for more information on how these locations are estimated in an integrated Bayesian phylogenetic framework. B) Visualization of antigenic drift dynamics reconstructed using Bayesian MDS in a two-dimensional map. These patterns are inferred from the 2002 to 2011 subset of the influenza A/H3N2 dataset analyzed by Bedford et al. (2014). X and Y represent the first and second antigenic dimensions. The contours represent the 80% HPD region for the node locations (both internal and external nodes). The colors range from green to blue for the lines, points and contours reflects the age between 2002 and 2011. This figure was made using SpreadD3 (Bielejec et al. 2016).

Another challenge may be that the trait is only indirectly observed through an assay. This is the case for influenza antigenic evolution, which is often assessed through pairwise measurements of cross-reactivity between influenza strains using the hemagglutination inhibition (HI) assay. The influenza virus population continually evolves in antigenic phenotype to escape host immunity in a process known as antigenic drift, which explains the continual need to update vaccine formulations. To capture this process from an incomplete table with noisy HI measurements (HI titres), Smith et al. (2004) proposed the use of multidimensional scaling (MDS) techniques to position viruses in a two-dimensional cartographic map such that the distance in the lower-dimensional space best fits the HI assay titres. More recently, Bedford et al. (2014) adopted a Bayesian formulation of MDS (Oh and Raftery 2001) that models the observed differences (HI titres) to be centered around their cartographic expectation with a Gaussian error (Fig. 3). As a prior on the unknown location parameters, their approach resorts to the phylogenetic Brownian diffusion process, which leads to an explicit connection between antigenic evolution and genetic relatedness. Bedford et al. (2014) apply this approach to HI data from all human influenza lineages, A/H3N2, A/H1N1, B/Victoria and B/Yamagata, and show that A/H3N2 evolves faster and in a more punctuated fashion than other influenza lineages. More recently, these differences in antigenic evolution, coupled to the nature of human behavior in seasonal influenza epidemiology, were shown to drive differences in migration rate and hence also epidemic success (Bedford et al. 2015).

Although the concept of phylogenetic Brownian diffusion was adopted by the comparative approach to specifically accommodate phylogenetic dependence among traits, the question may arise as to how much dependence really needs to be taken into account. In other words, to what extent does the phylogeny explain similarity among taxa traits? In pathogen phylodynamics, this question may apply to virulence or infection traits, which can also be heavily impacted by the host environment. Specifically, for chronic infections such as HIV-1 and HCV, the comparative framework is being deployed to determine to what extent the viral genotype can control for the rate of progression or infection outcome, sometimes leading to mixed conclusions (Alizon et al. 2010; Vrancken et al. 2014a; Hartfield et al. 2014; Hodcroft et al. 2014). Among the different approaches available to test or quantify this “phylogenetic signal,” Pagel’s $\lambda$ (Pagel 1999a) is commonly used. Conceptually, this parameter scales internal node heights of a tree, on which Brownian evolution of traits are being modeled, in such a way that any trait correlation scenario can be accommodated from the estimated phylogeny down to a star-like tree (completely independent taxa traits). The BEAST framework we highlight here incorporates Pagel’s $\lambda$ estimator and therefore supports simultaneous estimation of evolutionary history and trait phylogenetic signal (Vrancken et al. 2014a).

In the framework of emerging infectious diseases, a similar question relates to host shifts and what determines the sensitivity to viral infection in the new host. Longdon et al. (2011) examined this in great detail for three host-specific sigma viruses in *Drosophila* and measured viral titres upon experimentally infecting 51 *Drosophila* species with each of these viruses. Using a phylogenetic mixed model (PMM), the authors found that the host phylogeny could explain most of the variation in viral replication and persistence between different host species. A PMM finds its analog in mixed modeling in quantitative genetics, where phenotypes of individuals related by a pedigree are partitioned into additive genetic (heritable) and residual (nonheritable) components (Henderson 1984; Lynch and Walsh 1998; Housworth et al. 2004). The implementations essentially differ in their specification of the correlation structure of the heritable components: a relationship matrix for pedigrees versus a matrix of shared common ancestry in a phylogeny. It is interesting to note that estimates of phylogenetic signal through Pagel’s $\lambda$ find a similar degree of phylogenetic association of sensitivity to sigma virus infection compared to a standard phenotypic trait as wing size in *Drosophila* (Vrancken et al. 2014a). In addition to the sensitivity to infection following host shifts, it may also be important to predict how much harm the pathogen will cause in the new hosts. Virulence may be considered as a direct consequence of pathogen replication, but also the host may be an important determinant. To examine how virulence may vary across new host species, Longdon et al. (2015) carried out a large cross-infection experiment of *Drosophila* C virus in 48 species of Drosophilidae. In this case as well, PMM analyses showed that changes in virulence, which can be extremely large, were highly predictable from the host phylogeny (Longdon et al. 2015).

## Covariates of Sequence and Trait Evolution

Although many phylodynamic hypotheses can be addressed through the analysis of genetic data, trait data, or their combination, additional data in the form of covariates may be required to further dissect the dynamic forces that determine the diversity of epidemiological and phylogenetic patterns. Integrating such data in phylodynamic approaches can serve two purposes: better informing reconstructions and identifying which covariates explain the evolutionary or epidemiological process. In this section, we discuss a number of examples to highlight recent methodological advances to achieve these goals for sequence evolution and trait evolution (tree diffusion processes).

**Figure 4.**
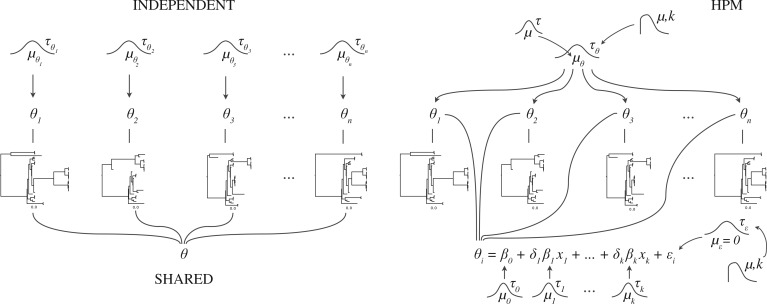
Contrasting models with completely linked and unlinked parameters to hierarchical modeling without and with fixed effects. Traditionally, two competing approaches were used when performing Bayesian inference to estimate parameters from a potentially large number of partitions, strata, or individuals: total evidence, where all the data across strata are pooled or shared to estimate a single parameter of interest, and unconditionally independent partitioning, where each stratum requires the estimation of a completely independent set of parameters. The latter is associated with independent prior specification on every parameter. Hierarchical phylogenetic models (HPMs) offer a middle ground between these two extremes by sharing a hierarchical prior distribution over all parameters with estimable mean and variance, which are drawn from hyperpriors. Edo-Matas et al. (2011) propose an HPM that employs a Bayesian mixed effects model that pools information across patients, affording more precise individual-patient parameter estimates when the data are sparse for a patient, but also allowing estimation of the effect of patient groups or continuous covariates.

## The Tempo of Sequence Evolution

The rate of evolution in different viral populations may be affected by their host environment, either through varying selective dynamics or through an impact on the replication rate. Although independent rate estimates could in theory be used to investigate this, a sparse heterochronous sequence sample from each population generally leads to uncertain evolutionary estimates which complicates formal statistical testing. In the context of HIV-1 evolution in different patient groups, Edo-Matas et al. (2011) proposed the use of a Bayesian hierarchical phylogenetic model (HPM) to pool information across patients and improve estimate precision for patient-specific viral populations, while still allowing for evolutionary rate differences between the individual populations. Hierarchical modeling was first introduced in phylogenetics to share information across alignment partitions (Suchard et al. 2003). By applying independent parameters that share a hierarchical prior distribution with unknown estimable hyperparameters, HPMs hold a middle ground between independent and shared parameter estimation (Fig. 4).

Following the terminology of ANOVA and regression models, the specification of hierarchical priors can model random effects on the evolutionary response variable. In order to test the impact of patient groups defined by disease progression and host genetic status, Edo-Matas et al. (2011) further extend the HPM approach by incorporating fixed effects for $N$ covariates, arriving at the following general form:

(1) $$\text{log}\theta_{i}=\beta_{0}+\delta_{1}\beta_{1}x_{i,1}+\ldots +\delta_{N}\beta_{N}x_{i,N}+\epsilon_{i},$$

where $\theta_{i}$ is the evolutionary response variables in patient $i$, ${\beta}_{0}$ is an unknown grand mean, ${\beta}$ is the estimated effect size of covariate $x$, ${\delta}$ is a binary indicator that tracks the posterior probability of the inclusion of covariate $x$ in the model and $\epsilon_{i}$ are independent and normally distributed random variables with mean 0 and an estimable variance. The specification of indicator variables (${\delta}$) implements a variable selection procedure analogous to the approach aimed at reducing the number of rate parameters in discrete diffusion matrices; although it was considered to be an approach for increasing statistical efficiency in the latter context, it is used here as a model averaging approach that effectively integrates over all possible combinations of covariate inclusion. For this and all other applications of variable selection we highlight, we note that covariate or fixed effect support values can be readily computed in the form of Bayes factors based on the posterior and prior odds for their inclusion. By applying this approach to within-host HIV-1 data sampled from different patient groups, Edo-Matas et al. (2011) not only demonstrate significant shrinkage of the estimator variance, but they also provide support for faster viral evolutionary rates in patients that progressed to AIDS more rapidly.

A similar need for shrinkage and hypothesis testing emerged in a study on the evolutionary consequences of host switching in bat rabies viruses in the Americas (Fig. 2) (Streicker et al. 2012). Although considerable variation in rabies evolutionary rates was noticeable among lineages associated with different hosts on this longer evolutionary time scale (Streicker et al. 2012), accurate quantification remained difficult due to limited sequence samples and their variation across different host species. The authors therefore constructed an HPM over the evolutionary rate parameters at the third codon position — as a proxy for synonymous evolution — in 21 independent bat rabies virus lineages. The fixed effects in their full model included physiological (basal and torpid metabolic rate), environmental (climatic region: temperate vs. tropics/subtropics) and ecological traits (coloniality and seasonal activity). Also in this case, HPM estimates proved to be less sensitive to stochastic noise associated with sampling error, and the authors were able to show an accelerated rate of molecular evolution in subtropical and tropical bats compared with temperate species. The association between geography and the tempo of evolution was explained by climate-associated differences in seasonality in bat activity and virus transmission.

The examples above consider categorical or continuous covariates for independent intrahost or interhost viral populations, but covariates may also represent categorical branch assignments in a phylogeny (conditionally independent evolutionary lineages). Vrancken et al. (2014b) took this into account in an examination of HIV-1 evolutionary rate differences within and between hosts in a known transmission chain. In this case, fixed effects were modeled as branch assignments that distinguish the transmitted lineage from other branches within specific hosts constituting the transmission chain. Random effects — in terms of possibly different rates for each branch — were modelled according to an uncorrelated relaxed clock process (following Drummond et al. (2006a)). This mixed effects modeling approach demonstrated a significantly slower rate for the transmitted lineage, which confirms earlier observations of evolutionary rate differences within and between hosts (Alizon and Fraser 2013) and provides support for the “store-and-retrieve” hypothesis in HIV transmission (Lythgoe and Fraser 2012; Fraser et al. 2014).

**Figure 5.**
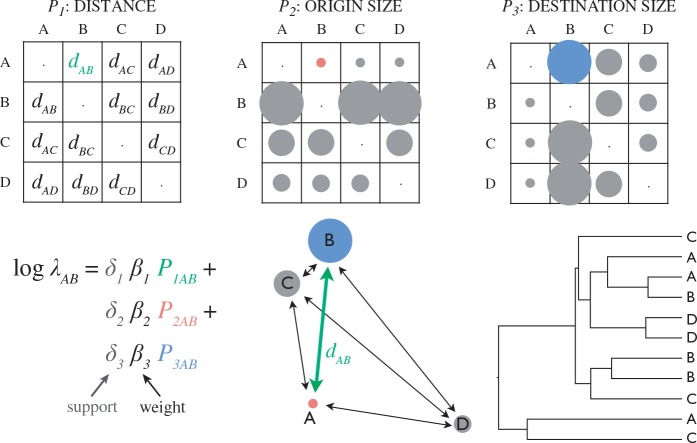
GLM extension of discrete phylogenetic diffusion and examples of covariates or predictors ($P$) in a spatial context. The GLM parameterizes each rate of among-location movement in the phylogeographic model as a log linear function of various potential predictors. For each predictor $P_{jXY}$ ($j \in \{1,2,3\}$; $X,Y \in \{A,B,C,D\}$), the GLM parameterization includes a coefficient $\beta_{j}$, which quantifies the contribution or effect size of the predictor (in log space), and a binary indicator variable $\delta_{j}$, that allows the predictor to be included or excluded from the model. Since predictors are essentially matrices of pairwise measurements, location-specific measurements such as population size or density are separated into origin or destination size.

## Tree Diffusion Processes

By extending the covariate modeling approach for a particular evolutionary parameter to a matrix of transition rate parameters defining a CTMC process, Lemey et al. (2014) developed an approach to simultaneously reconstruct spatiotemporal history and identify which combination of covariates associates with the pattern of spatial spread. This extension of the discrete phylogeographic diffusion approach (Lemey et al. 2009a) shares the generalized linear model (GLM) formulation for fixed effects introduced above, in this case by parameterizing each rate of among-location movement in the phylogeographic model — typically denoted as the $ij$-th elements ($\Lambda_{ij}$) of the transition rate matrix $\Lambda$ — as a log linear function of various potential covariates as following:

(2) $$\log\Lambda_{ij}=\beta_{1}\delta_{1}x_{i,j,1}+\beta_{2}\delta_{2}x_{i,j,2}+\ldots +\beta_{N}\delta_{N}x_{i,j,N},$$

where ${\beta}$ and ${\delta}$ represent the same parameters as before. In this case, a single covariate, also referred to as predictors in Lemey et al. (2014), is a flattened vector of quantities corresponding to entries in the $i$ to $j$ rate matrix $\mathbf{x}_{n} = (x_{1,2,n}, \ldots x_{K-1,K,n})^{'}$. In a phylogeographic setting, these predictors may, for example, represent a matrix of pairwise geographic distances or population sizes either at the origin or destination location (Fig. 5). As pointed out above, priors and posteriors for the inclusion probabilities (${\delta}$) can be used to express the support for each predictor in terms of BFs. Unlike the HPM or mixed effects modeling, the GLM parameterization does not consider random effects for the rates because of the difficulty to inform this large number of effects based on a single trait observation at the tree tips (but see (Trovão et al. 2015) for identifying exceptional random effects). The application of this approach to the seasonal dynamics of influenza H3N2 provided consistent support for air travel governing the global migration of the virus. We note that the GLM diffusion approach achieves both goals outlined in the introduction of covariates of evolutionary and epidemiological processes: it identifies the relevant covariates in the epidemiological process, but at the same time it helps to inform the ancestral reconstructions. For the influenza example, this may lead to more accurate reconstructions of the seasonal source-sink patterns, and in predictive modeling, the combination of genetic and air transportation data also outperformed both data sources in isolation (Lemey et al. 2014).

To demonstrate its generality, Faria et al. (2013) applied the GLM diffusion approach to host switching (jumps followed by successful transmission in the new host) and cross-species transmission (CST, spill-over in a viral clade associated with a dominant host) in bat rabies viruses (Fig. 2). These dynamics were separately investigated by Streicker et al. (2010) through standard ancestral reconstruction and structured coalescent analyses respectively, the latter resulting in rate estimates that were used to test several potential covariates as potential determinants of the CST dynamics. To simultaneously infer and test both host switching and CST in a single framework, Faria et al. (2013) made use of the flexibility of phylogenetic CTMC modeling to accommodate distinct host transition processes, parameterized by independent GLM diffusion models, on external and internal branches. To a large extent, this approach discriminates between recent CSTs, of which many are likely to result in dead-end infections, and the ancestral host shifts. The same predictors as used by Streicker et al. (2010) were considered for both branch-specific GLM diffusion parameterizations: host genetic distance, geographical range overlap and similarities in roost structures, wing aspect ratio, wing loading, and body size. The latter three morphological measurements represent approximations of foraging niche overlap in bats (Streicker et al. 2010). The full Bayesian analysis revealed that host similarity was strongly predictive of host jumping intensity, both for host switching and CST, whereas a modest support for geographical range overlap was only recorded for CST.

Although the primary purpose is likely to remain the identification of relevant covariates to integrate in tree diffusion processes, we note that the GLM diffusion model may offer the only practical solution to discrete ancestral reconstruction for traits with high-state spaces because it reduces the number of parameters to a linear function of the number of predictors rather than a quadratic function of the number of states. High-state spaces still challenge likelihood calculations, but massively parallel computations offer considerable speed-up in such cases, and practical solutions are now in place both in terms of hardware (e.g., GPU cards) and software to support phylogenetic likelihood computations on such hardware (e.g., BEAGLE) (Suchard and Rambaut 2009; Ayres et al. 2012; Baele and Lemey 2013).

## Data Integration to Elucidate Transmission Dynamics

In this section, we discuss how data integration is being considered to help uncover tree-generative processes in phylodynamics. We make a distinction between the reconstruction of large-scale epidemic dynamics based on a limited sample from the pathogen population and the reconstruction of transmission trees from densely sampled pathogen genetic sequences to recover the chain of transmission in extensive detail. Much of this work attempts to relate the transmission dynamics inferred from genetic data to mathematical epidemiology, or even explicitly builds a bridge between pathogen genetics and how transmission dynamics are formalized in compartmental models. As opposed to coalescent models describing the merging of lineages backward in time starting from a small sample of the population until a common ancestor has been reached, compartmental models like the susceptible-infected-removed (SIR) model describe the dynamics of an entire population going forward in time. Modeling infectious disease dynamics with compartmental models allows the description of nonlinear time series of prevalence of infection and the number of susceptible hosts, and leads to important predictions about pathogen spread, for example the prevalence and duration of the epidemic. They also offer a framework to assess the potential impact of intervention strategies on the outcome of an epidemic.

## Reconstructing Large-Scale Epidemic Dynamics

Using time-varying coalescent models, phylogenies can be used to estimate how effective population sizes ($N_{e}$) change through time. This $N_{e}$ represents the size of an idealized population that loses or gains genetic diversity at the same rate as the census population from which the sample has been drawn. Although frequently applied as the only source of information about past population dynamics, the dynamics of $N_{e}$ have sometimes been contrasted against other data. For example, Biek et al. (2007) demonstrated that the expansion of rabies in the North American raccoon population shows similar dynamics to an epidemiological index that reflects the size of the area newly affected by rabies during each month of the outbreak.

Bennett et al. (2010) also report a correspondence between fluctuations in $N_{e}$ and case counts for dengue serotype 4 between 1981 and 1998 in Puerto Rico. However, when formally assessing this relationship, they identify an offset of about seven months in the cyclical dynamics. Using a similar post-hoc procedure, Faria et al. (2012) also find a lag of about five years between prevalence counts and the growth in $N_{e}$ over time for HIV-1 CRF02_AG in Cameroon. Although these studies illustrate opportunities for integrating time series covariates, they also point at complications in interpreting $N_{e}$ estimates as “effective number of infections.” Frost and Volz (2010) specifically address this issue and note that the rate of coalescence is primarily driven by new transmission — that is the incidence — and only indirectly by the number of infected individuals through sampling effects (Frost and Volz 2010).

By avoiding the concept of $N_{e}$, birth–death modeling represents an interesting alternative that is gaining popularity as a tree-generative process. Originally introduced in phylodynamics by Stadler et al. (2012), the stochastic linear birth–death process is parametrized by a rate at which infected individuals transmit and the rate at which they turn noninfectious (either by death or recovery). For a completely susceptible population, the ratio of these rates should in principle quantify the well-known $R_{0}$ parameter from mathematical epidemiology. Unlike the coalescent, birth–death models do not need to assume a small sample size from a large population because the sampling proportion is treated as a separate parameter in these models. Following the evolution in coalescent modeling, the standard birth–death model has also been extended to a nonparametric version that flexibly models varying infection rates (Stadler et al. 2013). Despite the flexible parameterizations of coalescent and birth–death modeling approaches, they have yet to be extended to allow the incorporation of covariates.

Arguably the most concrete step toward integrating covariates with tree-generative processes has been made by Rasmussen et al. (2011). The authors fitted a stochastic, nonlinear model of disease transmission to a combination of epidemiological data and phylogenetic coalescence events. This approach follows the SIR coalescent modeling by Volz et al. (2009) and resorts to particle MCMC to fit state-space models to genealogies in order to avoid the need for an analytical likelihood function. Simulations suggest that genealogical information alongside of time series data may improve the estimates if the time series data suffers from observation error or from variation in reporting practices. The ability to combine coalescent information with covariates, or contrast it against them, also offers the opportunity to investigate which ecological factors need to be considered by the coalescent model to appropriately capture pathogen population dynamics. In a study of dengue serotype I in southern Vietnam, Rasmussen et al. (2014) demonstrate that nonparameteric coalescent modeling does not reproduce the highly seasonal incidence patterns observed in hospitalization data. In this case, incorporating spatial structure was critical to recapitulate seasonal fluctuations consistent with the hospitalization data. This illustrates that the panmictic population assumption in coalescent modeling can be problematic, and that inferring temporal epidemic dynamics cannot always be divorced from population structure, which we discussed in the context of trait or structured coalescent inference. Intriguingly, the authors also show the importance of accounting for vector population, suggesting a general need to consider ecological complexities in pathogen epidemiology.

## Reconstructing Transmission Trees

In the first section of this review, we focused on the integration of time and location with genetic data in a general phylogenetic framework, but these sources of information are sometimes also combined in different ways when the aim is to reconstruct transmission trees. Recreating individual routes of transmission in infectious disease outbreaks is a challenging problem, but one of longstanding interest in infectious disease epidemiology that essentially goes back to the famous work by John Snow who traced the source of a cholera outbreak in London in 1854 (Snow 1855). A clear break with the inference of large-scale transmission dynamics is the requirement of a dense — if not complete — sampling from the pathogen population, at least for the initial developments in this direction.

Person-to-person transmission is frequently represented as a spanning tree, and various methods have therefore been proposed to find the spanning tree between sampled sequences that is most compatible with the genetic data, for example by minimizing a set of edge weights (see e.g., Jombart et al. (2011)). Cottam et al. (2008) introduce epidemiological data by first retrieving the set of transmission trees that are consistent with the available genetic data, and then evaluating these trees using data on their relative timings to identify the most plausible transmission history. A more formal integration of epidemiological and genetic data can be found in the work of Ypma et al. (2012), who apply their Bayesian procedure to temporal, geographical, and genetic data on poultry farms infected in an epidemic of avian influenza A (H7N7) in The Netherlands in 2003. Although genetic and epidemiological data arise from the same process, this approach assumes they are independent. To address this limitation, Morelli et al. (2012) develop an MCMC approach that integrates epidemiological data and pathogen sequences from infected hosts to estimate transmission trees and infection dates in a more coherent way.

Generally, disease dynamics are only partially observed, but methods based on transmission trees are not designed to handle large numbers of missing infections, and therefore require a dense sample of infected hosts from the outbreak. To remedy this, Mollentze et al. (2014) propose a generalization of the algorithm of Morelli et al. (2012) to allow its application to any directly transmitted disease and enable reconstruction of partially observed transmission trees as well as to estimate the number of cases missing from the sample. The extension specifically allows accommodation of a wide variety of spatial transmission patterns and allows multiple unobserved cases to arise anywhere in both space and time within the set of inferred transmissions. An application to endemic rabies virus in a province of South Africa shows that the method offers a better insight into the spatial epidemiological patterns (Mollentze et al. 2014). In related work, Jombart et al. (2014) propose a Bayesian framework that does not require all cases to be observed or assume a single introduction event at the origin of an outbreak. Their framework allows for the estimation of dates of infections, mutation rates, separate introductions of the pathogen, the presence of unobserved cases and the transmission tree, as well as the effective reproduction number over time, thereby overcoming the limitations of their previous method (Jombart et al. 2011) and of the methods of Ypma et al. (2012) and Morelli et al. (2012). The authors apply their method to the 2003 Severe Acute Respiratory Syndrome (SARS) outbreak in Singapore, providing new insights into the early stage of the epidemic.

## Recent Developments and Future Perspectives

In this review, we highlighted several aspects of data integration in phylodynamics with a particular focus on the connection between sequences and traits. As mentioned in the respective sections, the initial applications of relatively simple but computationally efficient Bayesian implementations of random walk models in the field of pathogen evolution and epidemiology focused on reconstructions of spatial spread. There are, however, important caveats that need to be considered for phylogeographic applications, in particular related to sampling. First, spatial coverage may be restricted to particular geographic areas which limits comprehensive understanding of the spatial spread dynamics. For example, although large genetic datasets have become available to study the global seasonal dynamics of all human influenza lineages, the sampling remains limited for Africa, Central America, the Middle East, and Russia (Bedford et al. 2015). This complicates assessment of their specific role in the influenza source-sink dynamics. In the discrete ancestral reconstruction approach, heterogeneity in sampling among the locations that can be represented in the analysis may also bias the results. Overrepresentation of location states is likely to be associated with high rates out of, or into, these specific states, and these transition rates will influence ancestral state probabilities in the phylogenies. Although subsampling may be used to investigate the sensitivity to sampling effects, it remains difficult if not impossible to remove sampling bias in these approaches. The structured coalescent methods we discuss as alternative approaches are less sensitive to sampling bias because they take the diversity in each location or “deme” into consideration, and may therefore have a more promising future in phylogeographic studies. In the continuous diffusion approach, the parameterization does not have such a direct connection with the sampling, but other restrictions are important to consider in this framework. Despite the ability to relax the assumption of a constant diffusion rate, the process still assumes a relationship between dispersal and geographic distance, which may provide a poor fit to pathogens that disperse through modern human mobility. In addition, standard Brownian diffusion considers distances in Euclidean space and therefore ignores the spherical nature of the globe (but see Bouckaert (2015)). Alternatively, Barton et al. (2013) discuss the extension of the coalescent to spatially structured populations, where populations are not disjointly subdivided, but instead are distributed across a spatial continuum. The authors point out that in such a setting, the system of stochastic ordinary differential equations known as Kimura’s stepping stone model (Kimura 1953) has no solution in two spatial dimensions, as the system of coalescing random walks which describes the genealogy converges to a system of Brownian motions that will never meet.

Although the examples we discussed generally involve a single trait, there are no particular limitations on the number of traits that can be incorporated in a single analysis. Trovão et al. (2015) leverage this capacity to examine the contributions of different hosts to the spatial spread of avian influenza H5N1. Specifically, they jointly infer discrete host switching among members of different avian families/superorders and dispersal in continuous space underlying the H5N1 expansion across Eurasia. Although this study models the host and spatial dynamics as independent processes, it leads to host-specific summaries of spatial spread and testable differences among them. In addition to combining multiple traits, the possibility to incorporate covariates in discrete diffusion processes also prompts exploration of more detailed ecological information in pathogen evolution and epidemiology. In a spatial context, cheap and mobile global positioning systems are now widely adopted in the recording of infectious disease spread, but also the variables that are associated with geo-located disease data (e.g., environmental, infrastructural, and socio-economic) are increasingly being characterized in great detail and distributed as publicly available datasets (see e.g., http://www.worldpop.org.uk). In addition, particular forms of animal trade or human mobility are extensively documented, or they can be modeled based on proxies such as the movement of marked banknotes (Brockmann et al. 2006), and anonymized mobile phone call records (González et al. 2008).

The example of trait combinations (Trovão et al. 2015) also raises the question of how correlations can be measured formally between different data types. This is a particularly pertinent question for phenotypic traits in evolutionary biology, which explains correlations as the result of genetic constraints or selective effects. The multivariate diffusion approach naturally models and estimates correlation among continuous traits, but for different data types (e.g., binary and multinomial ordered, or unordered data) additional modeling is required. Building on the phylogenetic threshold model (Felsenstein 2005), Cybis et al. (2015) demonstrate how to efficiently infer the evolution of all types of traits, and the correlations among them, through latent liability modeling in the BEAST framework. One of their applications to pathogens targeted the phenotypic correlation among the amino acid sites of the antigenic epitopes of the influenza hemagglutin surface protein, which plays an important role in antigenic drift dynamics. They find strong correlations among 11 sites in epitope A and B, including all sites that have been experimentally shown to be responsible for evolving antigenic novelty (Koel et al. 2013). Such methodologies open up new research opportunities at the interface of genotype and phenotype in pathogen evolution, and in conjunction with antigenic (Bedford et al. 2014) and integrated genetic and human mobility modeling (Lemey et al. 2014) in the same Bayesian framework, this may lead to holistic approaches in influenza phylodynamics for example. The latent liability or threshold model finds its nonphylogenetic roots in a model that goes back to Sewall Wright (Wright 1934), and has been used regularly in the pedigree analysis of discrete traits (Gianola 1982). So, in addition to the PMM we discussed in the context of trait heritability, this highlights another explicit link between phylogenetics and quantitive genetics.

Further in the phenotypic context, other assumptions than the constant variance also require attention when modeling trait evolution via Brownian motion along a phylogeny. Assuming a zero-mean displacement, for example, postulates that a single phenotypic value randomly increases or decreases each generation, which can only be expected for phenotypes undergoing random genetic drift or fluctuating directional selection. Under other forms of natural selection however, traits may evolve toward some optimal value. To model such consistent selection toward a single optimum trait value, the Ornstein-Uhlenbeck (OU) model has been proposed as a mean-reverting extension of Brownian motion (Hansen 1997). Although this has proven useful to identify stabilizing selection regimes, a central tendency model does not offer a general solution to appropriately fit the complexity in trait evolution. Further advances in Bayesian trait evolutionary modeling may provide the flexibility to model displacements in traits along tree branches as a generalized stochastic process that allows the data to inform the degree of complexity required in the process.

Data integration in phylodynamic modeling is also transforming the inference of transmission dynamics. Although this is still somewhat in its infancy in the analyses of large-scale transmission dynamics based on coalescent or birth–death models, the possibility of using genetic data and time series data in tandem has been clearly demonstrated (Rasmussen et al. 2011), as well as the need to incorporate environmental stochasticity to accurately capture disease dynamics in some cases (Rasmussen et al. 2014). These developments are generally divorced from the sequence evolutionary process and still await their implementation in the commonly used Bayesian statistical software packages like BEAST (Drummond et al. 2012). Although BEAST implements a wide range of tree-generative processes, including time-variable coalescent and birth–death models, these have not yet been connected to covariate or time series data. Related to this, however, ongoing work in coalescent modeling acknowledges that sampling times may probabilistically depend on effective population size (Karcher et al. 2015), and preferential sampling is now being taken into account by modeling the sampling times as an inhomogeneous Poisson process dependent on effective population size.

Developments in transmission tree reconstruction have already been pursuing data integration for a longer time, and they are evolving toward methods that consider the likelihood of observing sequence and epidemiological data for a given transmission tree, and increasingly try to accommodate missing data. Many of these developments are however scattered in the field, and at least for the sequence evolutionary modeling, they could also benefit from an implementation in an integrated statistical framework. In addition, more complex relationships between phylogenies and transmission trees need to be taken into account (akin to gene/species tree modeling, Vrancken et al. (2014b)), in particular when within-host evolution plays an important role. Different aspects of within and between host evolution are not always easily reconciled, and this has been identified as one of the challenges in phylodynamic inference (Frost et al. 2015).

Accounting for nonvertical evolution represents another major challenge as all the methods we have discussed rely on a strictly bifurcating evolutionary process. Nonvertical evolution can be a prominent evolutionary force in many pathogen populations, for example in the form of recombination and reassortment in viruses and horizontal gene transfer in bacteria. Recombination analyses in viruses, for example based on the identification of tree incongruence, generally aim at avoiding the impact of recombination in downstream phylogenetic inferences (Martin et al. 2011). However, an alternative approach may be to explicitly accommodate nonvertical evolution through an ancestral recombination graph, which simultaneously describes vertical and nonvertical evolutionary events (Hudson 1983). Although the graph is a well-known model in coalescent inference, it has only been recently introduced as an explicit structure for analyzing phylogenetic data in BEAST (Bloomquist and Suchard 2010), and further modeling and computational development is required to promote its widespread use.
